# Modeling PAH Mixture Interactions in a Human In Vitro Organotypic Respiratory Model

**DOI:** 10.3390/ijms25084326

**Published:** 2024-04-13

**Authors:** Victoria C. Colvin, Lisa M. Bramer, Brianna N. Rivera, Jamie M. Pennington, Katrina M. Waters, Susan C. Tilton

**Affiliations:** 1 Department of Environmental and Molecular Toxicology, Oregon State University, Corvallis, OR 97331, USA; 2 OSU/PNNL Superfund Research Program, Oregon State University, Corvallis, OR 97331, USA; 3 Pacific Northwest National Laboratory, Richland, WA 99352, USA

**Keywords:** mixtures, polycyclic aromatic hydrocarbons, chemical interactions, 3D in vitro models, lung cells

## Abstract

One of the most significant challenges in human health risk assessment is to evaluate hazards from exposure to environmental chemical mixtures. Polycyclic aromatic hydrocarbons (PAHs) are a class of ubiquitous contaminants typically found as mixtures in gaseous and particulate phases in ambient air pollution associated with petrochemicals from Superfund sites and the burning of fossil fuels. However, little is understood about how PAHs in mixtures contribute to toxicity in lung cells. To investigate mixture interactions and component additivity from environmentally relevant PAHs, two synthetic mixtures were created from PAHs identified in passive air samplers at a legacy creosote site impacted by wildfires. The primary human bronchial epithelial cells differentiated at the air–liquid interface were treated with PAH mixtures at environmentally relevant proportions and evaluated for the differential expression of transcriptional biomarkers related to xenobiotic metabolism, oxidative stress response, barrier integrity, and DNA damage response. Component additivity was evaluated across all endpoints using two independent action (IA) models with and without the scaling of components by toxic equivalence factors. Both IA models exhibited trends that were unlike the observed mixture response and generally underestimated the toxicity across dose suggesting the potential for non-additive interactions of components. Overall, this study provides an example of the usefulness of mixture toxicity assessment with the currently available methods while demonstrating the need for more complex yet interpretable mixture response evaluation methods for environmental samples.

## 1. Introduction

Traditional chemical toxicity assessments tend to focus on studying the effects of a single chemical, but this is a great underestimation of the typical exposure scenario [1]. Organisms are most commonly exposed to chemical mixtures which can be varied and complex depending on their location, time of exposure, chemical source, and other factors resulting in unique health effects [2]. The US Environmental Protection Agency (EPA) and National Institute of Environmental Health Sciences (NIEHS) have recognized the risk of chemical mixture exposure on human health and the need for addressing methods in mixture toxicity assessment [3,4,5]. A major limitation in mixture toxicity assessment is the ability to adequately represent an exposure for testing. Three main methods have been developed for addressing mixture toxicity including whole mixture, sufficiently similar mixtures, and component-based approaches [5,6]. The whole mixture approach consists of testing a whole extract collected from environmental sampling to assess the specific toxicity of the environmental exposure. While whole mixture toxicity assessments provide the most useful data for specific exposure scenarios, they are limited by the availability of the whole mixture. The US EPA addresses possible concerns over whole mixture data calling for a consideration of the similarity of the collected mixture to the real exposure, stability of the mixture after collection, and potential of human or ecological exposure to the collected mixture [5]. When whole mixture data are unavailable or cannot be obtained, representative or sufficiently similar mixture toxicity data may be considered for the risk assessment. Sufficiently similar mixtures are formulated based on environmental sampling to best represent the whole mixture and may be created based on component abundance, toxicity, environmental fate, biological effects, or chemical class [7,8,9,10]. Sufficiently similar mixtures reduce the mixture but should be validated to ensure that the formulated mixture is truly representative of the whole mixture toxicity [9,10]. The US EPA recommends an in-depth analysis of the mixture’s similarity to the whole mixture with considerations of component composition differences, component concentration differences, and the bioavailability of either mixture or its components [5]. Lastly, component-based approaches are most commonly used in mixture risk assessment due to the lack of whole mixture and sufficiently similar mixture data or the standardization for their approaches [1,5,11,12]. Component-based assessment consists of compiling available toxicity data on the components of the whole mixture based on the interactions and toxicological similarity of the components [1,13]. If there is a lack of sufficient component data, component responses may be estimated based on a reference chemical according to the component toxic equivalency factors (TEFs) [5].

Component-based mixture toxicity assessments require the selection of a modeling technique to combine component data and characterize the mixture response for a given endpoint. Three commonly used modeling techniques for investigating mixture toxicity include concentration addition (CA), generalized concentration addition (GCA), and independent action (IA) [12,14,15,16]. These methods characterize the mixture response on the assumption that the component responses combine in an additive manner, but the resulting model trend can be compared to the actual mixture response to determine the potential for a weaker (antagonistic) or stronger (synergistic) response [15,16,17]. CA is the simplest of the three methods with the main assumption that all components behave the same in the system with the same mechanism of action and thus can be considered dilutions of each other [14,15]. A limitation of this method is that all components are assumed to have similar slopes in their response curves therefore limiting the maximal estimated response of the mixture to that of the component with the lowest maximal absolute response [14,15]. GCA is a derivation of the CA model and accounts for components with less than maximal effects by incorporating component EC50 values and the endpoint maximal response [18,19]. While this method removes the maximal effect limitation of CA, it is limited by the need to calculate an EC50 value for all component response curves. IA was originally derived for probability assessments but has been further developed to utilize quantitative data [17]. The TEF approach, which has primarily been applied to CA or GCA, is recommended by the US EPA as a placeholder to fill missing data gaps [5]. However, most studies using a TEF approach only utilize the reference chemical response in proportion to the component TEFs which limits the scope of the assessment by assuming all components act with a similar mechanism of action [19,20]. Unlike CA and GCA, IA is designed to model data from components that may exert toxicity with dissimilar mechanisms of action and allows for the use of more diverse data without the need for component EC50 or maximal effect values [11,12,14,21]. In the present study, we apply a component-based approach with IA modeling for a mixture of polycyclic aromatic hydrocarbons (PAHs) due to data input requirements and unknown mechanisms of action for several compounds in the evaluated mixtures. Additionally, we apply an adapted TEF approach for IA modeling by scaling component concentrations based on their respective TEFs while still utilizing component responses to remove the assumption of a similar mechanism of action. This study demonstrates and discusses the usefulness and limitations of this method for component-based additivity modeling for chemical mixtures.

PAHs are a large class of chemicals consisting of two or more fused benzene rings [22,23]. They are common environmental air pollutants with natural and anthropogenic sources such as wildfires, incomplete fossil fuel combustion, wood preservation, tobacco smoking, etc. [24,25,26,27,28,29]. Organisms are typically exposed to PAHs in complex mixtures with their composition determined by nearby sources [29]. Many PAHs have shown potential for mutagenicity, genotoxicity, immunotoxicity, reproductive toxicity, and developmental toxicity, and 16 PAHs have been designated as priority pollutants by the US EPA. Several studies have found evidence that mixtures of PAHs may elicit unique toxicity as compared to single chemical exposures suggesting potential for differences in the mechanism of action between PAHs [30,31,32,33,34]. Due to the growing concern of increased or unique toxicity, there has been a push for more research on PAH mixture toxicity assessments and methods for evaluating PAH mixture toxicity for risk assessment. It is important to consider the relevance of the chosen model for risk assessment since data generated in a model more similar to the population of concern will result in fewer uncertainties. Humans may be exposed to PAHs by inhalation, ingestion, or dermal contact, and PAHs have been detected in most human tissues [22,35]. The lung is a major target organ for PAH toxicity where short term exposures have been shown to impact individuals with asthma, and the metabolism of PAHs has been shown to reduce lung function in individuals with no previous conditions as well as increase the chance of lung cancer [35,36,37,38]. Primary human lung cell cultures can be a valuable tool in PAH toxicity assessment by providing more human-relevant mechanistic and toxicity data for PAH exposures as compared to immortalized cell lines or in vivo animal models. Organotypically cultured primary human lung cells have been demonstrated to have many similarities with the in vivo human lung including cellular differentiation and basal transcript expression levels [39,40]. Additionally, this model has previously been utilized for evaluating differential transcript expression after PAH exposures [30,41,42].

This study assesses the additivity assumption for PAH mixtures by investigating the combined effects of mixture components compared to the mixture response. Primary human bronchial epithelial cells (HBECs) cultured at the air–liquid interface (ALI) are used to investigate PAH mixture effects across multiple biomarkers of PAH exposure and toxicity. The mixture response is assessed based on the additivity assumption using the IA modeling of the individual component responses with and without TEF adjustments for the studied biomarkers after the optimization of model parameters for PAH mixture selection, exposure time, and dose range. Overall, this study provides guidance on the best practices for mixture toxicity assessment, provides further evidence on PAH mixture interactions, and shows the need for more research on the area of PAH mixture toxicity regarding the component mechanism of action and additivity.

## 2. Results

The overall goal of this study is to assess component toxicity, the mechanisms of action, and additivity in environmentally relevant PAH mixtures. In order to model the additivity of mixture components, it is necessary to first optimize parameters to determine the most appropriate PAH mixture, biomarkers, timepoints, and concentration ranges to utilize for IA modeling in the ALI-HBEC model. Multiple endpoints, including those associated with cytotoxicity, barrier integrity, xenobiotic metabolism, oxidative stress, and DNA damage, were evaluated in the ALI-HBECs after treatment with two environmentally relevant PAH mixtures across a range of concentrations and timepoints to select the optimal parameters for mixture interaction studies. Lactate dehydrogenase (LDH) leakage was used as an indicator of cytotoxicity, and transepithelial electrical resistance (TEER) was used as a functional measure of barrier integrity in ALI-HBECs. Differential expressions of *CYP1A1*, *CYP1B1*, *ALDH3A1*, *GSTA*, *HMOX1*, *NQO1*, *GJA1*, *TJP2*, and *DDB2* were used to evaluate transcriptional biomarker responses based on previous research which found these markers to be predictive of PAH carcinogenicity [30,43]. For additivity modeling, ALI-HBECs were either treated with a single PAH mixture, ToxMix, or with each of the individual PAH components across a range of concentrations for 24 h. Transcriptional biomarkers related to barrier integrity, xenobiotic metabolism, oxidative stress, and DNA damage were used for additivity modeling to characterize trends in the mixture response curves compared to component PAH responses. Finally, the outputs of two IA models were compared to the mixture response to assess component additivity and the potential for component interactions.

### 2.1. Evaluation of PAH Mixtures in ALI-HBECs

**Figure 1:**
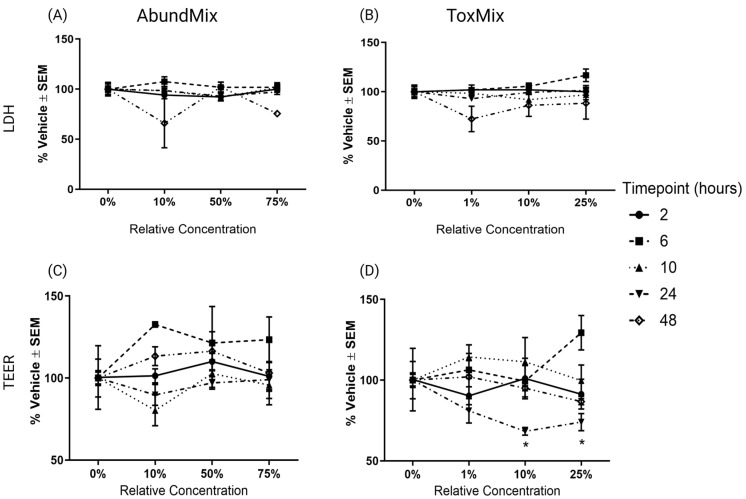
Cytotoxicity and barrier integrity from PAH mixture exposures as measured by LDH leakage and TEER, respectively. ALI-HBECs (*n* = 3) were apically treated with ToxMix or AbundMix for 2, 6, 10, or 24 h in DPBS with a 1% DMSO vehicle. (**A**) LDH leakage after AbundMix (75%, 50%, or 10%) exposure, (**B**) LDH leakage after ToxMix (25%, 10%, or 1%) exposure, (**C**) TEER after AbundMix (75%, 50%, or 10%) exposure, and (**D**) TEER after ToxMix (25%, 10%, or 1%) exposure. Data points represent the average % change normalized to the vehicle control. Error bars represent the standard error of the means. Asterisks indicate 640 level of significance (* *p*_adj_ < 0.05; one-way ANOVA with Dunnett’s post hoc test compared to the vehicle control). ToxMix (10%) at 24 h (*n* = 2) only. Created with BioRender.com (accessed on 10 April 2024).

To evaluate differences in cytotoxicity, barrier integrity, and transcriptional biomarker responses between two PAH mixtures, ALI-HBECs were exposed to ToxMix (25%, 10%, or 1%) or AbundMix (75%, 50%, or 10%) over 2, 6, 10, 24, or 48 h. There is no evidence of increased cytotoxicity from AbundMix or ToxMix at any concentration or timepoint tested (Figure 1A,B). Barrier integrity was only significantly (*p*_adj_ < 0.05) decreased by ToxMix exposures at or above 10% relative concentration at 24 h (Figure 1C,D). 

**Figure 2:**
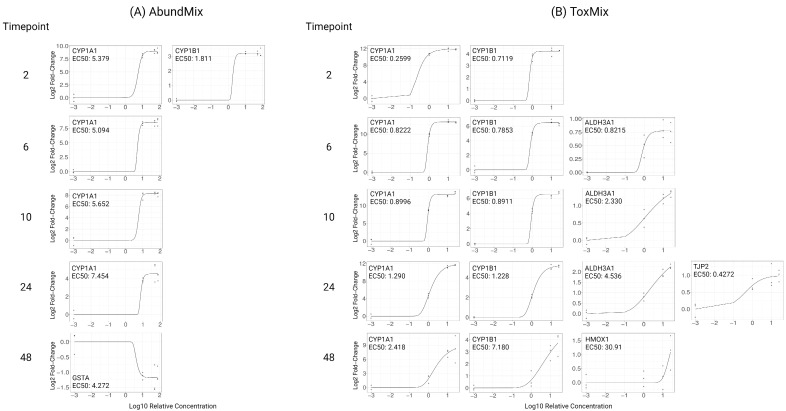
Significantly altered transcriptional biomarkers from PAH mixture exposure as measured by qPCR. ALI-HBECs (*n* = 3) were apically treated with (**A**) AbundMix (75%, 50%, or 10%) or (**B**) ToxMix (25%, 10%, or 1%) for 2, 6, 10, 24, or 48 h in DPBS with a 1% DMSO vehicle. Data points represent individual replicate responses. Regression lines represent the fitted model curve for the data. EC50 = half maximal effective concentration represented as relative concentration. Significance was determined as at least one concentration having a significant difference (*p*_adj_ < 0.05) from the vehicle control as evaluated by a one-way ANOVA with Dunnett’s post hoc test. Created with BioRender.com (accessed on 10 April 2024).

**Figure 3:**
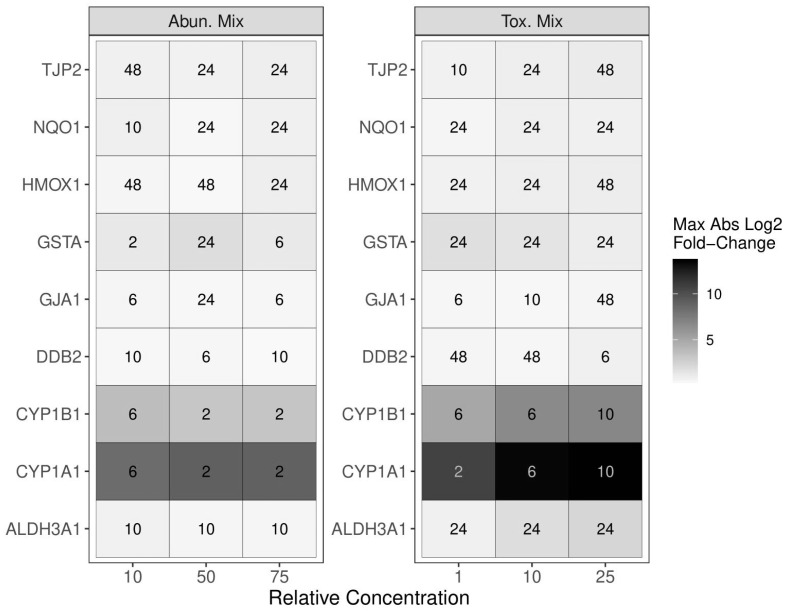
A summary of the differential transcriptional expression by AbundMix (**left**) and ToxMix (**right**) exposure. ALI-HBECs (*n* = 3) were apically treated with ToxMix (25%, 10%, or 1%) or AbundMix (75%, 50%, or 10%) for 2, 6, 10, 24, or 48 h in DPBS with a 1% DMSO vehicle. Numbers in boxes represent the timepoint in hours of the maximal fold change occurrence for each gene and mixture relative concentration. The color represents the magnitude of the maximal fold change at the designated timepoint for each gene and mixture relative concentration.

Transcriptional biomarkers for *CYP1A1*, *CYP1B1*, *ALDH3A1*, *GSTA*, *HMOX1*, *NQO1*, *GJA1*, *TJP2*, and *DDB2* were evaluated across concentration and time for each PAH mixture. Dose–response curves were fitted for all significantly (*p*_adj_ < 0.05) differentially regulated transcripts and their EC50 values calculated (Figure 2). AbundMix significantly (*p*_adj_ < 0.05) altered the expression of *CYP1A1*, *CYP1B1*, and *GSTA* in a dose-dependent manner for at least one timepoint with the most sensitive genes being *CYP1A1* and *CYP1B1* (Figure 2A). ToxMix significantly (*p*_adj_ < 0.05) altered expression for *CYP1A1*, *CYP1B1*, *ALDH3A1*, *HMOX1*, and *TJP2* in a dose-dependent manner for at least one timepoint with the most sensitive genes being *CYP1A1*, *CYP1B1*, and *ALDH3A1* (Figure 2B). AbundMix and ToxMix appeared to reach saturation in gene expression by the highest relative concentration (75% and 25%, respectively) for most genes that had a significant response. ToxMix resulted in a significant (*p*_adj_ < 0.05) alteration of more transcripts, had a larger maximal absolute change, and was more potent for most genes and timepoints. The timepoint of the maximal absolute fold change differed among mixtures, concentrations, and genes, with the most common timepoint being 24 h for both AbundMix and ToxMix (Figure 3), and ToxMix also demonstrated a higher maximal fold change for most evaluated genes.

### 2.2. Selecting Mixture Parameters for Additivity Modeling

There were three criteria for biomarker data to be considered in the selection of the mixture of interest for additivity modeling: (1) the endpoint showed significance (*p*_adj_ < 0.05) for at least one tested concentration, (2) the data fit a dose–response curve model, and (3) an EC50 could be calculated from the dose–response curve. Data that met these criteria can be seen in Figure 2. Due to the increased response in the number of significant transcripts, maximal absolute response, and potency, as seen in Figure 2 and Figure 3, ToxMix was chosen as the mixture of interest for additivity modeling. Mixture component exposure times and concentrations were selected to increase interpretability and decrease the complexity of the additivity model. Lower concentrations (10%, 5%, 1%, and 0.5%) were used to avoid transcript response saturation and place more emphasis on the exponential portion of the dose–response curve. The 24 h timepoint was chosen since this was the most common timepoint showing a maximal transcript response between ToxMix and AbundMix exposures (Figure 3). 

### 2.3. Evaluation of PAH Mixture Components in ALI-HBECs

To evaluate mixture component additivity and potential component interactions, cytotoxicity, barrier integrity, and transcriptional biomarker responses were evaluated from ALI-HBECs exposed to ToxMix (10%, 5%, 1%, or 0.5%) or an individual component (10%, 5%, 1%, or 0.5%) for 24 h. There is no evidence of elevated cytotoxicity (Figure S1A) or decreased barrier integrity (Figure S1B) after 24 h treatment with ToxMix or any of the ToxMix components at the concentrations tested. 

**Figure 4:**
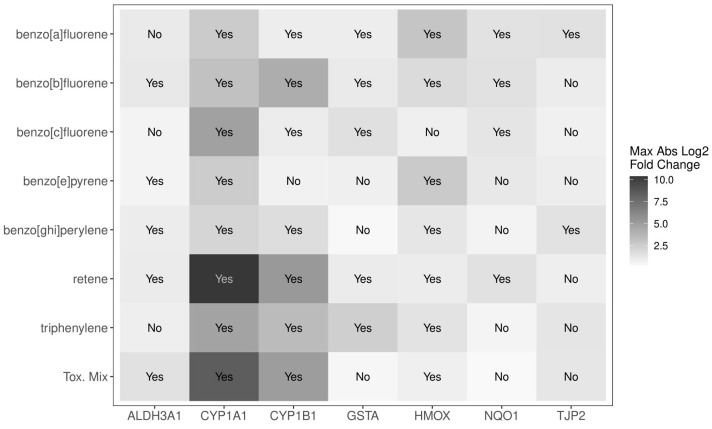
A summary of the differential transcriptional expression for ToxMix and individual component exposures. ALI-HBECs (*n* = 4) were apically treated with ToxMix, retene, BaF, BbF, BcF, triphenylene, BghiP, or BeP (10%, 5%, 1%, or 0.5%) for 24 h in DPBS with a 1% DMSO vehicle. “Yes” indicates that at least one concentration was significant (*p*_adj_ < 0.05) from the vehicle control for that treatment and gene control as evaluated by a one-way ANOVA with Dunnett’s post hoc test. “No” indicates no significance from the vehicle control at any concentration for that treatment and gene. The color represents the magnitude of the maximal fold change for that treatment and each gene over all concentrations.

Treatment with either ToxMix or a component over 24 h had a significant (*p*_adj_ < 0.05) result in seven of the nine tested genes including *ALDH3A1*, *CYP1A1*, *CYP1B1*, *GSTA*, *HMOX1*, *NQO1*, and *TJP2* (Figure 4). Retene was the most active of the individual components when exposed alone resulting in the highest absolute magnitude of change among all individual components for *CYP1A1* and *CYP1B1* and showing significance (*p*_adj_ < 0.05) in six out of seven transcripts. BaF and BbF were the next most active components when exposed alone also resulting in significant (*p*_adj_ < 0.05) alteration in six out of seven transcripts. *CYP1A1* was the most sensitive gene to PAH exposure with a significant (*p*_adj_ < 0.05) differential expression by all treatments.

### 2.4. Evaluating Mixture Additivity 

**Figure 5:**
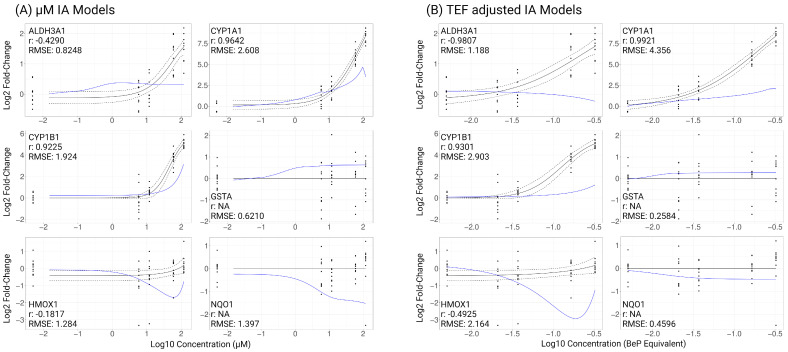
Independent action (IA) models characterizing ToxMix response of transcriptional biomarkers using (**A**) µM concentration of components represented as log10 µM concentration and (**B**) TEF-adjusted concentration of components represented as log10 BeP equivalent concentration. Cells (*n* = 4) were apically treated with ToxMix, retene, BaF, BbF, BcF, triphenylene, BghiP, or BeP (10%, 5%, 1%, or 0.5%) for 24 h in DPBS with 1% DMSO vehicle. Black solid line indicates mean ToxMix response, black dots indicate individual sample responses for ToxMix treatment, and black dotted lines indicate standard error of ToxMix response. Blue line indicates IA model of ToxMix response based on individual component data. RMSE represents root-mean-square error between modeled response and ToxMix response curve, and r represents Pearson correlation between modeled response and ToxMix response curves. Created with BioRender.com (accessed on 10 April 2024).

IA was utilized for additivity modeling based on model criteria relating to submaximal responses in the dose–response data from component and ToxMix exposures and the uncertainty in the mechanisms of action for many mixture components. To model an additive response from ToxMix, we applied the IA modeling method with transcriptional biomarker results from individual ToxMix component exposures using either µM component concentrations or TEF-adjusted component concentrations. We required a minimum of two significant (*p*_adj_ < 0.05) observations per gene, compound, and concentration for a treatment to be included in additivity modeling. *CYP1A1* for 10% BeP exposure did not meet this requirement and was removed from further analyses. For a gene to be included in additivity modeling, we required at least three components to result in a significant (*p*_adj_ < 0.05) response at any treatment concentration. ToxMix was not required to show a significant response. *TJP2* only had a significant (*p*_adj_ < 0.05) alteration by BaF and BghiP exposure and was removed from further analyses. A total of six of the nine evaluated genes were included in IA modeling. To evaluate model performance, we used the Pearson correlation coefficient (r) to assess the similarity of trends in the fitted curves between the model response and the ToxMix response and used the RMSE to assess similarity between the average response for the predicted IA model compared to ToxMix. The IA models using µM concentrations of the components (without TEFs) resulted in *HMOX1* having a weak correlation (|r| < 0.3), *ALDH3A1* having a moderate correlation (0.3 < |r| < 0.5), and *CYP1A1* and *CYP1B1* having a strong correlation (|r| > 0.5) with the actual ToxMix response (Figure 5A). Assessing the error of the modeled response by the RMSE shows *CYP1A1* having large error (RMSE > 2), *CYP1B1*, *HMOX1*, and *NQO1* having moderate error (1 < RMSE < 2), and *ALDH3A1* and *GSTA* having small error (RMSE < 1). The IA models using the TEFs of components resulted in *HMOX1* having a moderate correlation and *ALDH3A1*, *CYP1A1*, and *CYP1B1* having a strong correlation with the actual ToxMix response (Figure 5B). Assessing the error of the modeled response by the RMSE shows *CYP1A1*, *CYP1B1*, and *HMOX1* having large error, *ALDH3A1* having moderate error, and *GSTA* and *NQO1* having small error. There was no evidence of differential expression across dose for *GSTA* or *NQO1* after ToxMix exposure, and a correlation coefficient could not be calculated for either IA model methods. According to the Pearson correlation, the IA model that accounts for component TEF values better described the mixture response curve trends. According to the RMSE, the IA model using µM concentrations of the components without TEFs better described the mixture response. Neither IA model outperforms the other, and there are clear biases for each method. The TEF-adjusted IA model curves tend to follow the trend of the component with the highest TEF value, BeP in this case, and the µM (non-TEF) IA model curves tend to follow the trend of the most abundant component, retene being the most abundant in this case. Both IA model methods also tended to underestimate the ToxMix response for most genes which could be evidence for chemical interactions or potential synergism occurring in the actual ToxMix response. 

## 3. Discussion

### 3.1. Differential Expression of Transcriptional Biomarkers by PAH Mixtures

This study assessed additivity for an environmentally relevant PAH mixture using two IA models, with and without the inclusion of TEFs, to evaluate the predicted response of individual components compared to the mixture response in primary HBECs cultured at the ALI. Two environmentally relevant PAH mixtures were evaluated for their bioactivity and potential for use in additivity modeling. We found the mixture formulated based on toxicity metrics to be the most bioactive in human lung epithelium based on the differential expression of nine transcriptional biomarkers known to be altered by PAH exposure and predictive of PAH carcinogenicity [30,43]. There is an abundance of the literature researching the mechanisms of PAH toxicity with many studies finding PAHs, both single chemical and mixture exposure, leading to alterations of several cellular pathways such as barrier integrity, inflammation response, cell proliferation, cell cycle signaling, oxidative stress response, xenobiotic metabolism, DNA damage response, and immune response [30,44,45,46]. Genes related to oxidative stress, DNA damage, and inflammation response pathways tend to be up-regulated, while genes related to barrier integrity tend to be down-regulated [30,32,33,44,45,47,48]. Genes related to the metabolism of xenobiotics such as cytochrome P450s may be up- or down-regulated depending on the chemical or concentration of exposure [30,49]. 

Many genes play an important role in PAH toxicity whether it be for the activation or detoxification of xenobiotics. Several genes studied here have been shown to be differentially expressed by PAH exposure in previous studies, and we investigated the effects of two PAH mixtures on these common biomarkers of PAH exposure including *CYP1A1*, *CYP1B1*, *GSTA*, and *ALDH3A1* which have been demonstrated to mediate PAH metabolism and are biomarkers of PAH exposure [38,50,51,52,53,54,55]. Many individual PAHs and PAH mixtures similar to the ones studied here have been shown to induce *CYP1A1* and *CYP1B1* expression and protein activity, including retene, acenaphthene, BghiP, wildfire particulate matter (PM) extract, ambient air PM extract, coal tar extract (CTE), cigarette smoke, and various occupational mixture exposures. ToxMix, which is predominantly retene, was shown to be a more potent and efficacious *CYP1A1* and *CYP1B1* inducer with a lower EC50 at all timepoints and a larger magnitude of change when compared to AbundMix. However, there is evidence that some PAHs can inhibit these enzymes such as BeP, acenaphthene, and occupational mixture exposures [30,43,44,46,47,49,54,55,56,57,58,59,60,61,62,63]. In the current study, HBECs grown at the ALI were exposed to two PAH mixtures and evaluated for the differential expression of transcriptional biomarkers across dose and time. *GSTA* has previously been shown to be differentially expressed by both individual and mixture PAH exposures [30,43]. While there exists a large data gap surrounding modifications of the alpha group by PAHs, studies focusing on other GSTs, such as pi, mu, or theta GST groups, show a similar induction of the *GST* mRNA levels [56,64]. In the present study, AbundMix was the only mixture to result in a significant (*p*_adj_ < 0.05) change in *GSTA* expression with a decrease in expression after 48 h. The calculated EC50 for *GSTA* at the 48 h timepoint is lower than for *CYP1A1* showing an increased potency at this timepoint; however, *GSTA* was only significantly altered at a single timepoint compared to *CYP1A1* which was significantly induced at four out of five timepoints. Previous studies have shown individual and mixture PAH exposures to differentially regulate the expression of *ALDH3A1* with many mixture exposures such as CTE and tobacco smoking increasing the expression. The direction of the differential expression from individual PAH exposure appears to rely on the PAH being tested with many PAHs inducing expression and others, such as phenanthrene, inhibiting expression [30,43,45,46]. In the current study, only ToxMix significantly (*p*_adj_ < 0.05) induced the expression of *ALDH3A1* at 6, 10, and 24 h. The increase in expression was moderate with the maximal fold change at the 24 h timepoint, but the EC50 tended to be large depending on the timepoint giving a similar potency to *CYP1A1* and *CYP1B1* but a maximal absolute response more similar to *GSTA*. Overall, toxicity- and abundance-based PAH mixtures differentially express transcriptional biomarkers related to xenobiotic metabolism with high efficacy and potency, and *CYP1A1* was the most sensitive biomarker to PAH mixture exposure.

PAHs have been shown to induce oxidative stress in human cells, and there exists antioxidant mechanisms to combat excess oxidative stress and reactive oxygen species (ROS) production [32,44,48]. To assess the oxidative stress response from exposure to two PAH mixtures, we investigated changes in transcriptional expression for *HMOX1* and *NQO1*, two known antioxidant genes [32,54,65,66,67]. Previous studies have demonstrated *HMOX1* differential expression after PAH exposures with most research showing an induction such as from organic particulate matter (PM) extract, diesel exhaust PM extract, oil burn PM extract, and several individual PAH exposures [32,44,48,49,68]. In the present study, *HMOX1* was only significantly (*p*_adj_ < 0.05) induced by ToxMix at the 48 h timepoint. *NQO1* has been demonstrated to be differentially expressed by PAH exposures with most studies showing an induction of the gene from exposures such as PM extract, smoking, CTE, naphthalene, and phenanthrene [43,46,56,59]. However, *NQO1* was not altered after AbundMix or ToxMix exposure in the present study at any timepoint or concentration. While the toxicity-based PAH mixture moderately altered the expression of transcriptional biomarkers for the oxidative stress response, *HMOX1* was the least sensitive in this study.

The integrity of the lung epithelial layer is crucial in maintaining lung health. However, PAHs have been shown to disrupt epithelial and barrier integrity through a reduction in tight and gap junction function [30,33,45,47]. We investigated the effects of two PAH mixtures on tight and gap junction function through the differential expression of *GJA1* and *TJP2* [69,70,71,72,73]. *GJA1* expression has been shown to be altered in mouse lung cells by PAH mixture exposure consisting of compounds commonly found in cigarette smoke [33,47]. In the present study, AbundMix and ToxMix did not significantly alter *GJA1* expression at any timepoint or concentration. *TJP2* has been shown to be induced by organic extracts of urban air PM which included BeP and BghiP [74]. In the present study, *TJP2* was only significantly (*p*_adj_ < 0.05) induced by ToxMix exposure at the 24 h timepoint. *TJP2*, while not as sensitive as the xenobiotic metabolism biomarkers, was more sensitive to PAH mixture exposure than the oxidative stress response biomarkers. Overall, the toxicity-based PAH mixture moderately altered the expression of transcriptional biomarkers for tight junction integrity. 

Many PAHs have been shown to cause DNA damage and are associated with increased carcinogenicity [75]. We investigated the potential for DNA damage by the two PAH mixtures by evaluating the differential expression of *DDB2*, a DNA damage response biomarker [76,77]. *DDB2* has previously been shown to be induced by PAH extract from nut roasting PM, and several genes related to DNA damage response have been shown to be differentially expressed by individual and mixture PAH exposure [43,49,53,58,63,78]. However, in the present study, AbundMix and ToxMix did not significantly alter *DDB2* expression at any timepoint or concentration.

### 3.2. Differential Expression of Transcriptional Biomarkers by Mixture Components

We further evaluated the response of the transcriptional biomarkers to PAH mixture and component exposure for mixture additivity modeling. There exists little to no research on the toxicity, alteration of transcriptional biomarkers, or mechanism of action for several of the components. Retene was the most bioactive component of ToxMix showing the largest maximal fold change in transcriptional expression especially for *CYP1A1* and *CYP1B1* as well as having a significant (*p*_adj_ < 0.05) response for all six genes considered for additivity modeling. Retene has been shown to greatly induce *CYP1A* expression with reliance on the aryl hydrocarbon receptor (AhR) in zebrafish [62,79]. BbF was the next most bioactive component with significance (*p*_adj_ < 0.05) in all six genes considered for additivity modeling and large inductions for *CYP1A1* and *CYP1B1*. Hawliczek et al. found that BbF greatly induced *CYP1A* expression in zebrafish, and responses from BbF exposure most likely occurred through interaction with the AhR [79]. While BeP has the largest assigned TEF among the ToxMix components with a value of 1, it was the least bioactive component with significant bioactivity for only three of the six genes considered for additivity modeling. Prior studies report that BeP increases *CYP1A1* expression or activity in rats and mice and interacts with glycine N-methyltransferase (GNMT) at a higher affinity than the AhR for CYP450 regulation [80,81,82]. BghiP exhibited moderate bioactivity compared to the other ToxMix components with significance (*p*_adj_ < 0.05) in four of the six genes considered for additivity modeling. Previous studies have found BghiP to have a higher affinity for GNMT compared to the AhR, not significantly inducing *CYP1A1* expression in rat and human liver cells [81,83]. While Cherng et al. found that BghiP did not significantly bind to the AhR or induce *CYP1A1*, they observed it did enhance AhR binding and *CYP1A1* expression in the human liver when co-exposed with benzo[a]pyrene suggesting a possible interaction of the two PAHs [83]. These data suggest some dissimilar mechanisms of action between the components in ToxMix. Specifically, BeP and BghiP have the potential to act through the GNMT pathway, retene and BbF most likely act through the AhR pathway, and BaF, BcF, and triphenylene have an unknown mechanism for transcriptional regulation.

### 3.3. Modeling Mixture Response Based on Component Additivity

While whole mixture or sufficiently similar mixture approaches are preferred, the most commonly used method for mixture risk assessment is a component-based approach due to the lack of whole mixture and sufficiently similar mixture data or the standardization for their approaches. The US EPA cautions the use of the component-based approach with large, complex mixtures given that the uncertainty in component interactions grows substantially with more components present [5]. Component-based approaches limit the assessment of complex interactions between chemicals of different classes but allow for more simplicity in the toxicity assessment which leads to more interpretable results and usefulness of the assessment. While more complex tools for mixture toxicity assessment have been created through the use of machine learning methods, there is a need for simpler, more interpretable methods to aid in faster integration into current risk assessments for regulatory decisions [4]. If there is not sufficient data for all mixture components for the endpoint of interest, component responses may be estimated from a reference chemical based on their TEF values, and the mixture toxicity assessment is conducted as if the mixture components are dilutions of the reference chemical. While the US EPA recommends using this approach to fill data gaps, there is debate over the usefulness of the TEF approach for PAH mixtures due to uncertainties in the component mechanisms of action [5,20,84]. Here, we apply a modified version of the component-based TEF approach by scaling component concentrations based on TEFs while still utilizing individual component responses as opposed to an estimated reference chemical response. This adaption should eliminate uncertainties in using reference chemical responses by not assuming all components are direct dilutions of each other with similar mechanisms of action. 

The mixture response can be characterized by the component data using an additivity model such as CA, GCA, or IA all of which assume that the components do not physically, chemically, or biologically interact and combine in an additive manner to create the mixture response. While CA has been a popular method for mixture risk assessments, many of its assumptions have been questioned including components having similar mechanisms of action and similar response curve slopes [85,86]. However, CA has shown to describe the additive mixture response well when components have a similar or mostly similar mechanism of action [87,88]. The GCA derivation of the CA method has been shown to give better additive mixture response characterization when components exhibited different response curve slopes [18,19]. The simplicity of CA and GCA is what makes these models the default methods used by regulators when only component data are available [89]. The TEF approach applied with CA and GCA models has also been shown to characterize mixture exposure well in some cases. Petry et al. demonstrated the usefulness of the TEF approach to characterize PAH mixture carcinogenicity, and Howard et al. demonstrated the TEF approach to characterize the mixture response as well as a GCA model when components have similar mechanisms of action but not when components have dissimilar mechanisms of action [19,90]. Unlike CA and GCA, IA does not assume that all components exert toxicity through a similar mechanism of action [11,12,14,21]. Originally, IA was used as a probability assessment for a certain biological endpoint, but a newer version has been derived to apply the model to more quantitative data [17]. IA has been shown to better characterize the mixture response when components have a dissimilar mechanism of action, while CA and IA have similar performances for components with similar mechanisms of action, and IA has been shown to overcome the limitation of characterizing only up to the maximal effect of the weakest compound seen in the CA model [17,34,91,92,93,94,95]. While IA may be more accurate in certain circumstances, CA is noted to give a more conservative evaluation for mixture toxicity and, thus, is considered more useful for regulatory decisions [14,89,96,97]. Additivity modeling with component-based approaches has been shown to have many limitations and errors in assumptions for PAH mixtures mainly involving the additivity of responses or effects assumption [31,98,99]. However, component-based methods compared to mixture effects have shown expected additive effects in some cases [17,98,100]. Previous studies have found IA models to characterize the mixture response well when components have dissimilar mechanisms of action but tends to describe a lower mixture response or incorrect trend of the mixture response curve when components have similar or mostly similar mechanisms of action [87,88,91,93,94,95,97,101]. Here, we also applied a modified TEF approach by scaling component concentrations to their respective TEFs. By applying this method, the modeled curve for the mixture response will be altered and may more closely resemble the observed mixture response curve. In the present study, both IA models, with and without TEFs, mainly underestimate mixture responses or provide an incorrect trend of the mixture response curve based on the RMSE and Pearson correlation metrics, respectively. There are two possible explanations for these differences from the mixture response: ToxMix components exert toxicity through potential component interactions resulting in a non-additive or synergistic toxicity of the mixture or may represent similar mechanisms of action. The incorrect curve trend characterized by the Pearson correlation could be explained by components of ToxMix acting through mostly similar mechanisms of action to alter the transcriptional expression of the evaluated biomarkers. However, as described above, there is evidence for some dissimilar mechanisms of action between ToxMix components with some having a high affinity for the AhR and others for GNMT. The incorrect characterization of the mixture response based on the RMSE metric could be explained by the components having non-additive interactions. It would appear that the components potentially interact in a synergistic manner. However, there is little research on how these PAHs may interact on a chemical or physical level. There exist several examples of the inability of CA, GCA, and IA to adequately characterize the mixture response as well as the hazards of chemical interactions such as when combining low- and high-molecular-weight PAHs demonstrating the importance of the mechanism of action and potential component interactions, as in this study [14,18,31,33,34,47,88,92,99,102,103]. In order to further address these hypotheses, more data would need to be collected for PAH alterations on transcriptional biomarkers, the mechanisms of action for differential transcriptional regulation, and the potential for chemical or physical interactions between compounds.

### 3.4. Best Practices for Additivity Modeling of PAH Mixtures

The findings from this study have highlighted some key considerations for PAH mixture toxicity assessments by component-based approaches. When evaluating the PAH mixture response, it is beneficial to consider the advantages and disadvantages for all methods and choose the most appropriate for the current mixture of interest. The endpoint of interest should be selected and fully understood prior to conducting toxicity assessments. In addition, the potential mechanisms of action for the mixture components need to be evaluated, taking into consideration how those mechanisms may affect the endpoint of interest. If using component-based approaches, the modeling method should also be chosen with consideration for component similarity, known interactions, the availability of component data, and the ability to collect new component data. The inclusion of the TEF concentration adjustment should also be taken into consideration if the component mechanisms of action are unknown or not well understood, but more research is needed to describe the utility of the adapted TEF approach with additivity modeling. Lastly, new modeling methods need to be formulated and validated to account for potential component interactions as opposed to strictly modeling additive responses. 

## 4. Materials and Methods

### 4.1. Mixture Formulation

Two PAH mixtures were previously formulated based on environmental air samples from a legacy creosote site impacted by wildfires [10]. Briefly, air samples were collected by low-density polyethylene stationary air samplers. Samples were concentrated to 1 mL and analyzed for 63 PAHs using an Agilent 7890B gas chromatograph with an Agilent 7000C triple quadrupole mass spectrometer (Agilent, Santa Clara, CA, USA). The Abundance Mixture (AbundMix) was formulated from 6 of the most abundant PAHs in the whole mixture: naphthalene, acenaphthene, 2-methylnaphthalene, 1-methylnaphthalene, fluorene, and phenanthrene, and the Toxicity Mixture (ToxMix) was formulated from the 7 most toxic PAHs in the whole mixture assessed by available and predicted toxicity metrics: retene, benzo[a]fluorene (BaF), benzo[b]fluorene (BbF), benzo[c]fluorene (BcF), triphenylene, benzo[e]pyrene (BeP), and benzo[ghi]perylene (BghiP) (Table S1). Both mixtures conserved environmentally relevant ratios of components (Table 1), and it is notable that they contain no overlap in chemical constituents. 

### 4.2. Primary Human Bronchial Epithelial Cell Maintenance and Exposure

Normal HBECs (Lonza, Basel, Switzerland) were expanded to passage 4 in PneumaCult Ex Plus medium (STEMCELL Technologies, Vancouver, BC, Canada) and transferred to 24-well plates with transwell inserts for differentiation at the air–liquid interface (ALI) using PneumaCult ALI medium (STEMCELL Technologies, Vancouver, BC, Canada). Cells were cultured at the ALI for 25 days at 37 °C and 5% CO_2_ with media changes every 2 to 3 days. On day 25, cells were rinsed with DPBS before apically treating with 25 uL of AbundMix (75%, 50%, or 10%, *n* = 3, Table S2a) or ToxMix (25%, 10%, or 1%, *n* = 3, Table S2b) in DPBS with a 1% DMSO vehicle for 2, 6, 10, 24, or 48 h or apically treated with 25 µL of ToxMix, retene, BaF, BbF, BcF, triphenylene, BeP, or BghiP (10%, 5%, 1%, or 0.5%, *n* = 4) in DPBS with a 1% DMSO vehicle for 24 h (Table S2b). Apical wash and basal media were collected and stored at −80 °C. Tissues were collected from inserts in an RLT lysis buffer with 1% β-mercaptoethanol using the Qiagen RNeasy Mini Kit (Qiagen, Hilden, Germany) and stored at −80 °C.

### 4.3. Transepithelial Electrical Resistance (TEER)

TEER was measured using an epithelial volt-ohmmeter (World Precision Instruments, Sarasota, FL, USA). The volt-ohmmeter was calibrated using a test electrode prior to the measurements. At time zero and the time of collection, DPBS was added to both apical and basal chambers and resistance was measured (ohms) for each insert. The results were adjusted for background resistance by subtracting the resistance measured from an empty insert.

### 4.4. Cytotoxicity

Lactate dehydrogenase (LDH) leakage was measured in media after treatment using a Pierce™ LDH Cytotoxicity Assay Kit (Thermo Fisher, Waltham, MA, USA) per the manufacturer’s protocol except half volumes of the basal media, reaction mixture, and stop solution were used. Cytotoxicity was calculated by subtracting the absorbance at 680 nm from the absorbance at 490 nm using a Synergy HTX plate reader (BioTek, Winooski, VT, USA). 

### 4.5. Real-Time Quantitative PCR (RT-qPCR)

RNA was isolated from tissues using the RNeasy Mini Kit (Qiagen, Hilden, Germany) per the manufacturer’s protocol and was quantified on a Synergy HTX plate reader equipped with a Take3 module (BioTek, Winooski, VT, USA). RNA quality was evaluated based on a 280/260 ratio. cDNA was synthesized using the SuperScript III First-Strand Synthesis Supermix for RT-qPCR (Invitrogen/Thermo Fisher Scientific, Waltham, MA, USA) per the manufacturer’s protocol except half volumes of 2X RT Reaction Mix and RT Enzyme Mix were used for a final volume of 10 µL. RT-qPCR reactions were performed using a BioRad CFX96 system (BioRad Laboratories, Hercules, CA, USA). The thermocycler was programmed for 1 cycle 95 °C for 1 min initial denaturing, 40 cycles 95 °C for 15 s denaturing, 60 °C for 30 s annealing/elongation, and a melt curve 65–95 °C/0.5° per 5 s for validating single product amplification. Human *CYP1A1*, *CYP1B1*, *ALDH3A1*, *GSTA*, *HMOX1*, *NQO1*, *GJA1*, *TJP2*, and *DDB2* levels were normalized to *PPIA* using the ΔΔCt comparative method with primers from Invitrogen (Table S3, Invitrogen, Waltham, MA, USA). 

### 4.6. Statistical Analysis

Three treatment replicates were used for timecourse exposures of AbundMix and ToxMix, and based on a power analysis to calculate the number of replicates needed to determine the statistical significance of endpoints, five replicates were used for the additivity modeling of ToxMix compared to individual component exposures. Outliers were identified and removed from the datasets prior to statistical analysis. Significant treatment effects for all assays were evaluated relative to the vehicle control using a one-way ANOVA with Dunnett’s post hoc test. A significance level cutoff was defined at an adjusted *p*-value of 0.05.

### 4.7. Dose–Response Analysis

Dose–response models for each treatment compound were fit to each gene analyzed by qPCR using the drc v3.0-1 R package [104]. Log-logistic, Weibull, Michaelis–Menten, asymptotic regression, and Gompertz models were fit to the data. Additionally, linear polynomial regression models (i.e., standard linear and quadratic regression models) were considered. The dose–response model with the smallest Akaike information criterion (AIC) was taken to be the optimal fitting model. If a polynomial model had the smallest AIC, we chose the best fitting dose–response model unless the polynomial AIC was smaller than the dose–response model by more than 2 [105]. The half maximal effective concentration (EC50) was identified for each non-polynomial fit curve. 

### 4.8. Independent Action Modeling

An IA model was used to model the ToxMix response for each gene at 24 h post exposure using two variations of component data. One variation used the µM concentration of components, and the other scaled components based on toxicity. Component concentrations were scaled based on their respective toxicity equivalency factors (TEFs, Table 2), and the resulting values are reported as BeP equivalent concentration. We used the IA model formulated in Labib et al. [17] with a modification to account for genes with compounds displaying up- and down-regulation as presented in Schüttler et al. [103] and given by Equation (1).

(1) $${log}_{2}{FC}_{mix}\left(c_{mix}\right)=\left[1-\prod_{i=0}^{n_{up}}\left(\frac{{log}_{2}FC\left(c_{i,up}\right)}{{maxlog}_{2}{FC}_{up}}\right)\right]\times {maxlog}_{2}{FC}_{up}+\left[1-\prod_{i=0}^{n_{down}}\left(\frac{{log}_{2}FC(c_{i,down})}{{maxlog}_{2}{FC}_{down}}\right)\right]\times {maxlog}_{2}{FC}_{down}$$

In this formulation, log_2_FC_mix_(*c*_mix_) is the modeled response of the mixture at a given concentration, log_2_FC(*c*_i_) of the response induced by the component, maxlog_2_FC is the maximum response, and up and down signify up- and down-regulation, respectively [17,103]. The Pearson correlation between the fitted models was calculated based on the observed mixture response and the IA model response to quantify the similarity of fitted curves between the observed and modeled trends and could only be calculated for data that fit a dose–response curve. The root-mean-square error (RMSE) between the mixture response and the modeled response was calculated to quantify the similarity between the observed and modeled data.

## 5. Conclusions

In summary, component-based additivity was assessed by IA modeling across multiple biomarkers for usefulness in characterizing the risk for representative PAH mixtures created from complex environmental samples for which limited data exist about component toxicity, including a lack of understanding of the mechanisms of toxicity. Biomarker responses associated with PAH toxicity and carcinogenicity were evaluated using a 3D organotypic lung model and assessed for component additivity using the IA modeling of component biomarker responses. Additionally, the use of TEF adjustments on component concentrations was evaluated for their utility in the IA model characterization of the mixture response after the optimization of model parameters for PAH mixture selection, exposure time, and dose range. We found that the IA modeling of component interactions adequately described the mixture response well in some cases but did not fully characterize the mixture response overall, generally underestimating toxicity and suggesting non-additive component interactions. Some differences between the IA models and mixture response may also suggest similar or mostly similar mechanisms of action between mixture components for the selected endpoints tested. This study demonstrates the limitations of the current additivity models and important considerations needed when conducting the additivity modeling of component data. In addition, this study shows the need for the further development of more complex yet interpretable mixture response evaluation methods for PAH mixtures representing the complexity of environmental samples. 

## Figures and Tables

**Table 1:** AbundMix and ToxMix components and relative ratios.

AbundMix		ToxMix	
Component	Percent of Mixture	Component	Percent of Mixture
Naphthalene	44%	Retene	68.6%
Acenaphthene	18.3%	Benzo[a]fluorene	16.8%
2-methylnaphthalene	15.9%	Benzo[b]fluorene	8.42%
1-methylnaphthalene	11.9%	Benzo[c]fluorene	4.21%
Fluorene	6.7%	Triphenylene	1.68%
Phenanthrene	3.16%	Benzo[e]pyrene	0.168%
		Benzo[ghi]perylene	0.0842%

**Table 2:** Toxicity equivalency factors (TEFs) for ToxMix components used for independent action modeling as reported in Messier et al. [106].

Compound	TEF
Retene	0.001
Benzo[a]fluorene	0.001
Benzo[b]fluorene	0.001
Benzo[c]fluorene	0.001
Triphenylene	0.001
Benzo[e]pyrene	1
Benzo[ghi]perylene	0.01

## Data Availability

Data are contained within the article and supplementary materials.

## Supplementary Materials

The following supporting information can be downloaded at https://www.mdpi.com/article/10.3390/ijms25084326/s1.
