# Supplementary material for: Modeling PAH Mixture Interactions in a Human In Vitro Organotypic Respiratory Model
Source: Int J Mol Sci. 2024 Apr 13;25(8):4326. doi: 10.3390/ijms25084326 (PMC11050152; doi:10.3390/ijms25084326)
Supplement: Supplementary file 1 [file ijms-25-04326-s001.zip › Colvin et al_Supplemental.pdf]

Supporting Information for Modeling PAH mixture interactions in a human in vitro 3D respiratory culture system

Victoria Colvin [1,2], Lisa M. Bramer [2,3] , Brianna N. Rivera [1,2], Jamie M. Pennington [1], Katrina M. Waters [2,3] and Susan C. Tilton [1,2]

1 Department of Environmental and Molecular Toxicology, Oregon State University, Corvallis, OR, USA; susan.tilton@oregonstate.edu (S.C.T.)

2 OSU/PNNL Superfund Research Program, Oregon State University, Corvallis, OR, USA

3 Pacific Northwest National Laboratory, Richland, WA, USA

Table of Contents:

Tables:	Page Number
S1: CAS#, purity, and supplier for compounds used to make AbundMix and ToxMix.	3
S2a: Component concentrations in AbundMix exposure solutions in a DPBS with 1% DMSO…	3
S2b: Component concentrations in ToxMix exposure solutions in a DPBS with 1% DMSO…	3
S3: Forward and reverse primer sequences used for RT-qPCR.	4
**Figures:**	**Page Number**
S1: Cytotoxicity and barrier integrity from PAH mixture and component exposures as…	5

Table S1. CAS#, purity, and supplier for compounds used to make AbundMix and ToxMix.

Compound	CAS#	Purity	Supplier
Naphthalene	91-20-3	99%	Sigma Aldrich
Acenaphthene	83-32-9	100%	AccuStandard
2-methylnaphthalene	91-57-6	98.4%	AccuStandard
1-methylnaphthalene	90-12-0	99.6%	AccuStandard
Fluorene	86-73-7	98.1%	AccuStandard
phenanthrene	85-01-8	99.5%	AccuStandard
Retene	483-65-8	97%	Santa Cruz Biotechnology
Benzo[a]fluorene	238-84-6	>98%	Santa Cruz Biotechnology
Benzo[b]fluorene	243-17-4	98.1%	AccuStandard
Benzo[c]fluorene	205-12-9	98%	TRC
Triphenylene	217-59-4	99.4%	AccuStandard
Benzo[e]pyrene	192-97-2	99.9%	AccuStandard
Benzo[ghi]perylene	191-24-2	98.9%	AccuStandard

Table S2a. Component concentrations in AbundMix exposure solutions in a DPBS with 1% DMSO vehicle.

	Concentration (µM)		
**Compound or Mixture**	**75%**	**50%**	**10%**
Naphthalene	585.2	390.1	78
Acenaphthene	243.2	162.1	32.4
2-methylnaphthalene	211	140.6	28.1
1-methylnaphthalene	158.2	105.5	21.1
Fluorene	90.2	60.2	12
phenanthrene	42.1	28.1	5.6
AbundMix	1329.9	886.6	177.2

Table S2b. Component concentrations in ToxMix exposure solutions in a DPBS with 1% DMSO vehicle.

	Concentration (µM)				
**Compound or Mixture**	**25%**	**10%**	**5%**	**1%**	**0.5%**
Retene	202.5	81.4	40.7	8.1	4
Benzo[a]fluorene	50	20	10	2	1
Benzo[b]fluorene	25	10	5	1	0.5
Benzo[c]fluorene	12.5	5	2.5	0.5	0.25
Triphenylene	5	2	1	0.2	0.1
Benzo[e]pyrene	0.5	0.2	0.1	0.02	0.01
Benzo[ghi]perylene	0.25	0.1	0.1	0.01	0.005
ToxMix	297.5	118.7	59.4	11.9	5.9

Table S3. Forward and reverse primer sequences used for RT-qPCR.

Gene	Direction	Sequence
PPIA	F	GCATACGGGTCCTGGCATCTTGTCC
PPIA	R	ATGGTGATCTTCTTGCTGGTCTTGC
CYP1A1	F	TCGGCCACGGAGTTTCTTC
CYP1A1	R	GGTCAGCATGTGCCCAATCA
CYP1B1	F	CCAACCTGCCCTATGTCCT
CYP1B1	R	CTGGATCAAAGTTCTCCGGG
ALDH3A1	F	TGTTCTCCAGCAACGACAA
ALDH3A1	R	AGGGCAGAGAGTGCAAGG
GSTA	F	CTGCCCGTATGTCCACCTG
GSTA	R	AGCTCCTCGACGTAGTAGAGA
HMOX1	F	CTCTGAAGTTTAGGCCATTG
HMOX1	R	AGTTGCTGTAGGGCTTTATG
NQO1	F	GGAGAGCACTGATCGTACTGGC
NQO1	R	GGATACTGAAAGTTCGCAGGG
GJA1	F	TGGTAAGGTGAAAATGCGAGG
GJA1	R	GCACTCAAGCTGAATCCATAGAT
TJP2	F	GGCCTACGACCCAGACTAC
TJP2	R	ACTCTTCGTTCGCTCTGCTTT
DDB2	F	ACCTCCGAGATTGTATTACGCC
DDB2	R	TCACATCTTCTGCTAGCACCG

(previously reported in Chang, Y., Siddens, L. K., Heine, L. K., Sampson, D. A., Yu, Z., Fischer, K. A., Löhr, C. V., & Tilton, S. C. (2019). Comparative mechanisms of PAH toxicity by benzo[a]pyrene and dibenzo[def,p]chrysene in primary human bronchial epithelial cells cultured at air-liquid interface. *Toxicology and Applied Pharmacology*, *379*, 114644. https://doi.org/10.1016/j.taap.2019.114644)

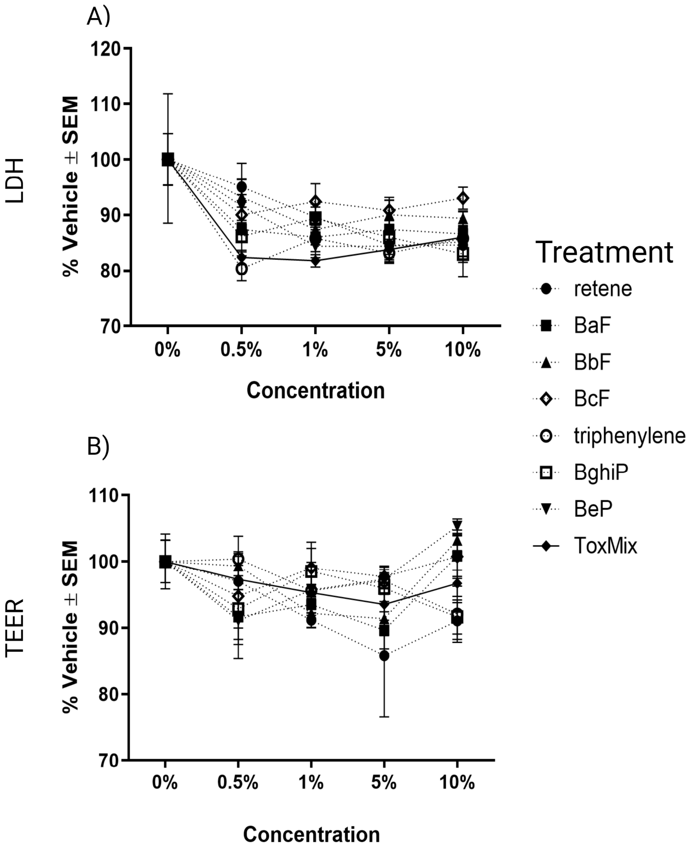

Figure S1. Cytotoxicity and barrier integrity from PAH mixture and component exposures as measured by LDH leakage and TEER, respectively. ALI-HBECs (n=4) were apically treated ToxMix, retene, BaF, BbF, BcF, triphenylene, BghiP, or BeP (10%, 5%, 1%, or 0.5%) for 24 hours in a DPBS with 1% DMSO vehicle. A) LDH leakage after ToxMix or component exposure B) TEER after ToxMix or component exposure. Data points represent the average % change normalized to the vehicle control. Error bars represent the standard error of the means. Significance was evaluated using a one-way ANOVA with Dunnett's post-hoc test compared to the vehicle control. Created with BioRender.com.
